# Effect of Process Parameters on the Microstructure and Properties of Cu–Cr–Nb–Ti Alloy Manufactured by Selective Laser Melting

**DOI:** 10.3390/ma16072912

**Published:** 2023-04-06

**Authors:** Jian Li, Zuming Liu, Huan Zhou, Shupeng Ye, Yazhou Zhang, Tao Liu, Daoyan Jiang, Lei Chen, Runxing Zhou

**Affiliations:** State Key Laboratory for Powder Metallurgy, Central South University, Changsha 410083, China

**Keywords:** Cu–Cr–Nb–Ti alloy, selective laser melting, process parameters, microstructure, properties

## Abstract

The fabrication of high-performance copper alloys by selective laser melting (SLM) is challenging, and establishing relationships between the process parameters and microstructures is necessary. In this study, Cu–Cr–Nb–Ti alloy is manufactured by SLM, and the microstructures of the alloy are investigated by X-ray diffraction (XRD), scanning electron microscope (SEM), and electron backscatter diffraction (EBSD). The effects of processing parameters such as laser power and scanning speed on the relative density, defects, microstructures, mechanical properties, and electrical conductivity of the Cu–Cr–Nb–Ti alloy are studied. The optimal processing window for fabricating Cu–Cr–Nb–Ti alloy by SLM is determined. Face-centered cubic (FCC) Cu diffraction peaks shifting to small angles are observed, and there are no diffraction peaks related to the second phase. The grains of XY planes have a bimodal distribution with an average grain size of 24–55 μm. Fine second phases with sizes of less than 50 nm are obtained. The microhardness, tensile strength, and elongation of the Cu–Cr–Nb–Ti alloy manufactured using the optimum processing parameters, laser power of 325 W and scanning speed of 800 mm/s, are 139 HV0.2, 416 MPa, and 27.8%, respectively, and the electrical conductivity is 15.6% IACS (International Annealed Copper Standard). This study provides a feasible scheme for preparing copper alloys with excellent performance and complex geometries.

## 1. Introduction

Cu–Cr–Nb alloys have excellent mechanical properties, electrical conductivity, and high-temperature stability. These alloys have broad application prospects in aerospace engines, nuclear power, and electrical power systems [1,2,3]. Cu–Cr–Nb alloys have attracted extensive attention since NASA [4] carried out research on them.

The solid solubility of the Cr and Nb atoms in the Cu–Cr–Nb alloys are small, and the atoms easily form the Cr_2_Nb phase with a high melting point, elastic modulus, and hardness [5,6]. It is difficult to effectively control the Cr_2_Nb phase by subsequent treatment, which seriously affects the comprehensive properties of the alloy. Cu–Cr–Nb alloys are traditionally prepared through melt-spinning, casting-deformation, and powder metallurgy. Strip Cu–2Cr–0.5Nb (at.%) alloy fabricated by melt-spinning has fine grains and a uniform composition [7]. Cu–Cr–Nb alloys fabricated by casting-deformation have excellent mechanical properties: the room-temperature tensile strength of Cu–0.47Cr–0.16Nb alloy can be as high as 453 MPa [8], and Cu–2Cr–1.35Nb–0.15Zr alloy can achieve a value of 126 HV in terms of microhardness [9]. However, the stored energy in the deformed alloy grains is high, and recrystallization will occur under high-temperature, softening the alloy, reducing its strength, and affecting its high-temperature service performance. Currently, the main powder metallurgy processes used for preparing Cu–Cr–Nb alloys are hot-press sintering (HPS) and spark plasma sintering (SPS). Cu–8Cr–4Nb (at.%) alloy with a high density can be fabricated by HPS [10], but the sintering temperature is high, reaching 1000 °C, resulting in a large size of the Cr_2_Nb phase in the sintered parts. The tensile strength of Cu–2Cr–1Nb alloy fabricated by SPS can reach 332 MPa [11]. The microhardness of Cu–2Cr–1.35Nb–0.15Zr (wt.%) alloy can reach a value of 133 HV, and the electrical conductivity is 74.6% IACS [12]. Parts fabricated by SPS are small in size, making it difficult for them to meet the requirements of large-sized components. Therefore, it is urgent to develop a process for preparing Cu–Cr–Nb alloys with a large size and excellent performance.

The cooling rate of SLM is fast, which can effectively refine grains and improve the comprehensive properties of alloys. SLM for as-built parts with complex geometries has been widely used in various metallic materials [13,14,15], such as titanium alloys [16,17], aluminum alloys [18,19], nickel-based superalloys [20,21,22], stainless steel [23,24], and other alloys. Recently, SLMed pure copper and copper alloys [25,26] have also been carried out. Copper alloys have a high laser reflectivity, and SLMed parts of Cu–Cr–Nb alloys have high porosity [27], which seriously affects the mechanical properties of the as-built parts. The hot isostatic press (HIP) treatment of as-built parts can effectively eliminate small pores [27,28] and improve the elongation of the alloy, but the tensile strength significantly reduces. SLM process parameters are optimized to improve the relative density of the fabricated parts, improving the alloy properties. High-density pure Cu [29] and Cu–Cr–Zr alloys [30] were obtained by optimizing process parameters such as laser power, scanning speed, and overlap spacing. Ma et al. [31] established a statistical model of the effect of process parameters on the relative density of as-built parts using the response surface method and fabricated parts with a relative density of 99.4%. Ren et al. [32] reported that laser power and scanning speed have a great influence on the relative density of the alloy. Cu–1.93Cr–0.74Nb (at.%) alloy manufactured using the optimum SLM process parameters after heat treatment has a tensile strength of 680 MPa and an electrical conductivity of 73.3% IACS [33]. Seltzman et al. [34] used SLM to fabricate a Cu–8Cr–4Nb (at.%) alloy with a tensile strength of 740 MPa and elongation of 20%. Ai et al. [35] optimized the laser power and exposure time of SLM to fabricate a Cu–Cr–Nb-Ce alloy with a relative density of 99.2%. The tensile strength of the alloy after heat treatment was 680 MPa.

Studies have shown that Ti can effectively improve the hardness and strength of Cu–Cr [36] and Cr–Nb [37] alloys. In this study, a new high-strength and high-conductivity Cu–Cr–Nb–Ti alloy based on Cu–Cr–Nb alloys was developed using the SLM process. The effects of laser power and scanning speed on the relative density, defects, grains, second phases, mechanical properties, and electrical conductivity of the alloy were studied. The SLM process parameters were optimized to fabricate Cu–Cr–Nb–Ti alloy with excellent performance. The obtained highly supersaturated solid solution lays the foundation for subsequent heat treatment to manufacture high-strength and high-conductivity Cu–Cr–Nb–Ti alloys.

## 2. Materials and Methods

### 2.1. Materials

**Figure 1:**
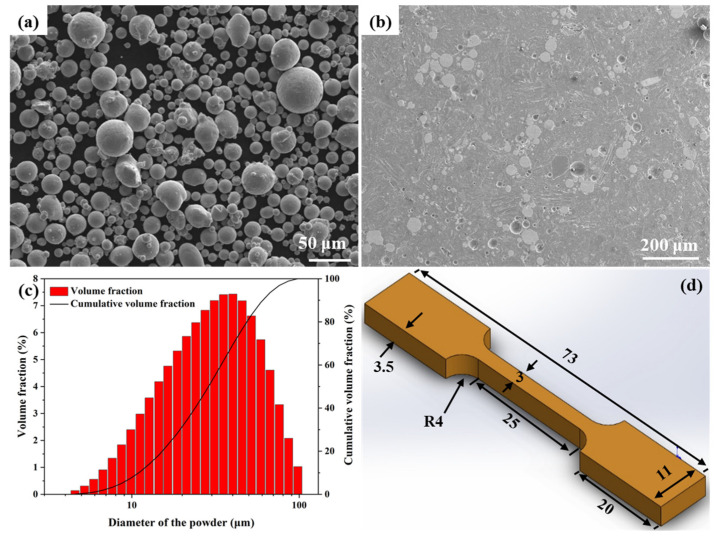
The Cu–Cr–Nb–Ti alloy powders and tensile sample: (**a**) powder morphology, (**b**) powder internal structure, (**c**) particle size distribution, and (**d**) tensile sample diagram.

The Cu–Cr–Nb–Ti powders were produced by argon atomization. The detailed preparation process was outlined in Ref. [38]. The chemical composition is presented in Table 1. The morphology and internal structure of the Cu–Cr–Nb–Ti alloy powders are shown in Figure 1a,b. The powders were spheroidal, and no pores were observed. The particle size distribution is analyzed by a laser particle size analyzer. Figure 1c shows the average particle size is 34.8 μm. *D*v (10) and *D*v (90) are 12.5 μm and 68.9 μm, respectively. The particle size distribution is centralized, which is conducive to uniform paving in the SLM process.

### 2.2. SLMed Cu–Cr–Nb–Ti Alloy

Cu–Cr–Nb–Ti alloy samples were fabricated using a SLM machine. Table 2 lists the laser power, scanning speed, and the number of as-built samples. The range of laser powers and scanning speeds is selected based on Cu–Cr–Nb alloys [32]. Other process parameters are specified in our previous work [32]: the layer thickness of 30 μm, the hatch spacing of 0.1 mm, the rotation angle between adjacent as-built layers of 67°, and the snake scanning strategy. The SLM process was conducted under an argon atmosphere. The volumetric energy density (VED) *E* (J/mm^3^) is expressed as:

(1) E=P/vht

where *P* is the laser power (W), *v* is the laser scanning speed (mm/s), *h* is the hatch spacing (mm), and *t* is the layer thickness (mm).

### 2.3. Microstructural Characterization and Performance Testing

The phase analysis of the alloy was carried out using XRD with a Cu Kα radiation source. The measurement was made within the 20°–80° range at a speed of 1°/min and a step size of 0.02°. XRD data were analyzed by Jade 6.5 software. The surface of the samples was observed using an optical microscope. The scanning electron microscope (SEM) was used to observe the defects and the second phase after etching for 40 s in a solution made of 25 mL ammonia water, 50 mL (NH_4_)_2_S_2_O_8_ solution, and 25 mL water. The size of the second phase was determined using Image-Pro Plus 6 software. Electron backscatter diffraction (EBSD) analysis was performed using the same SEM, and samples were etched by 70% H_3_PO_4_ and 30% water for 2 V and 9 s.

The density of the as-built samples was tested using the Archimedes density measurement method. The relative density (%) was calculated by dividing the density values of as-built samples by the density values of corresponding powders. The density values of powders (8.8559 g/cm^3^) were measured using the volumetric displacement method (ISO 12154:2014 [39]). A conductivity meter was used to test the electrical conductivity of the samples. Microhardness was measured using a microhardness tester with a load of 200 g and a dwell time of 15 s. The room-temperature tensile property was obtained using a universal testing machine at a stretching speed of 1 mm/min. The tensile sample diagram is shown in Figure 1d. All testing was repeated three times, and the average value was taken as the experimental result.

## 3. Results and Discussion

### 3.1. Relative Density and Defects

**Figure 2:**
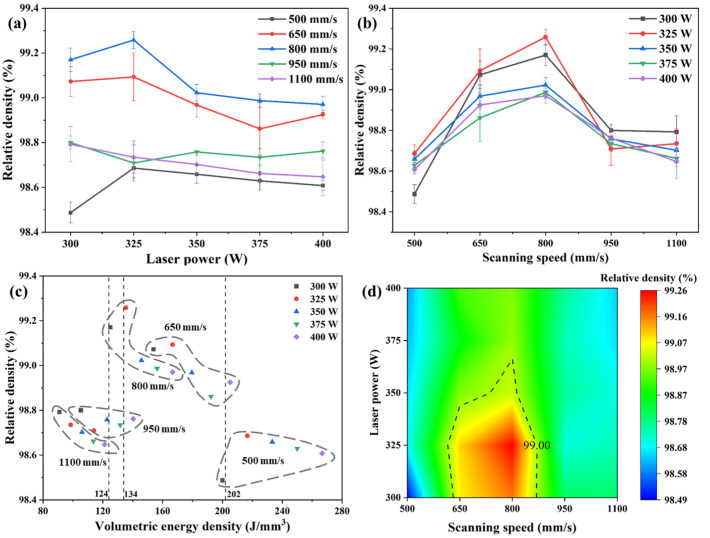
The relative density of Cu–Cr–Nb–Ti alloy manufactured using different process parameters: (**a**) the change curve of the relative density with the laser power under different scanning speeds, (**b**) the change curve of the relative density with the scanning speed under different laser powers, (**c**) the relationship between the VED and the relative density (symbols of the same color represent samples manufactured using the same laser power, and the dotted line frame represents samples manufactured at the same scanning speed), and (**d**) the process window of the Cu–Cr–Nb–Ti alloy (the process parameters of the as-built samples with a relative density greater than 99% are shown in the dashed frame).

Relative density is a quantitative index used to characterize defects such as pores and cracks comprehensively and is also an important evaluation index of the SLM process. In this article, relative density is used to comprehensively evaluate the formability of a Cu–Cr–Nb–Ti alloy. Figure 2 shows the effect of SLM process parameters on the relative density of the as-built Cu–Cr–Nb–Ti samples. Figure 2a shows the effect of laser power on relative density. It shows that there is a nonlinear relationship between relative density and laser power. At different scanning speeds, the effect of laser power on the relative density is quite different. When the scanning speed is 500–800 mm/s, with an increase in laser power from 300 to 400 W, the relative density of the as-built samples first increases and then decreases and is the highest when the laser power is 325 W. When the scanning speed increases to 950 mm/s, the laser power has no obvious effect on the relative density of the as-built samples, and the relative density fluctuates around 98.76%. When the scanning speed increases to 1100 mm/s, the relative density of the as-built samples decreases with an increase in laser power.

Figure 2b shows the effect of scanning speed on relative density. It shows that there is also a nonlinear relationship between relative density and scanning speed. When the scanning speed increases from 500 mm/s to 1100 mm/s, the relative density of as-built samples first increases and then decreases and is the highest when the scanning speed is 800 mm/s. According to the results in Figure 2a,b, the relative density of the as-built Cu–Cr–Nb–Ti sample prepared with a laser power of 325 W and scanning speed of 800 mm/s is the highest, reaching 99.26%. In summary, the effect of scanning speed on the relative density of Cu–Cr–Nb–Ti alloy as-built samples is greater than that of laser power. This may be different in the span of the parameter range. The span of the adjacent scanning speed range is greater than the span of adjacent laser powers in this study.

Figure 2c shows the relative density of Cu–Cr–Nb–Ti as-built samples prepared using different laser VEDs. The relative densities of as-built Cu–Cr–Nb–Ti samples manufactured with a similar VED are significantly different. The relative densities of samples P_300_V_800_ and P_350_V_950_ with a VED of about 124 J/m^3^ are 99.17% and 98.76%, respectively, with a difference of 0.41%. The relative density of sample P_325_V_800_ with a VED of 135 J/m^3^ is the highest, reaching 99.26%, while the relative density of sample P_375_V_950_ with a VED of 132 J/m^3^ is only 98.73%. The relative density of sample P_300_V_500_ with a VED of 200 J/m^3^ is 98.49%, which is significantly lower than that of sample P_400_V_650_ with a similar VED (a relative density of 98.93% with a VED of 205 J/m^3^). The effect of VED on relative density is influenced by laser power and scanning speed. There is little difference between the relative densities of the samples manufactured at the same scanning speed but different laser powers. The difference between the relative densities of the five samples manufactured at a scanning speed of 500 mm/s is 0.20%. The differences between the relative densities of the samples manufactured at scanning speeds of 650, 800, 950, and 1100 mm/s are 0.23%, 0.29%, 0.09%, and 0.15%, respectively. In addition, the relative densities of the five samples manufactured using the same laser power but different scanning speeds differ greatly. When the laser powers are 300, 325, 350, 375, and 400 W, the relative densities of the samples differ by 0.68%, 0.57%, 0.36%, 0.36%, and 0.36%, respectively. As per the above results, the effect of VED on the relative density is relatively complex, and VED can only be used as a reference parameter for the SLMed Cu–Cr–Nb–Ti alloy. This may be because VED can only be used to evaluate the laser energy input in the SLM process roughly but cannot be used to evaluate the impact of a single process parameter on the dynamic process of the molten pool. This dynamic process generates pores in the alloy, affecting the relative density of the Cu–Cr–Nb–Ti alloy.

Figure 2d shows the SLM process window of the Cu–Cr–Nb–Ti alloy. The combination of process parameters corresponding to a relative density greater than 99.0% (in the dotted box in Figure 2d) is regarded as the best process window. The SLM process window of the Cu–Cr–Nb–Ti alloy is significantly wider than that of the Cu–Cr–Nb alloy [32], which indicates that Ti microalloying can effectively broaden the process window of the Cu–Cr–Nb alloy system and is conducive to fabricating Cu–Cr–Nb–Ti as-built samples with high relative densities.

**Figure 3:**
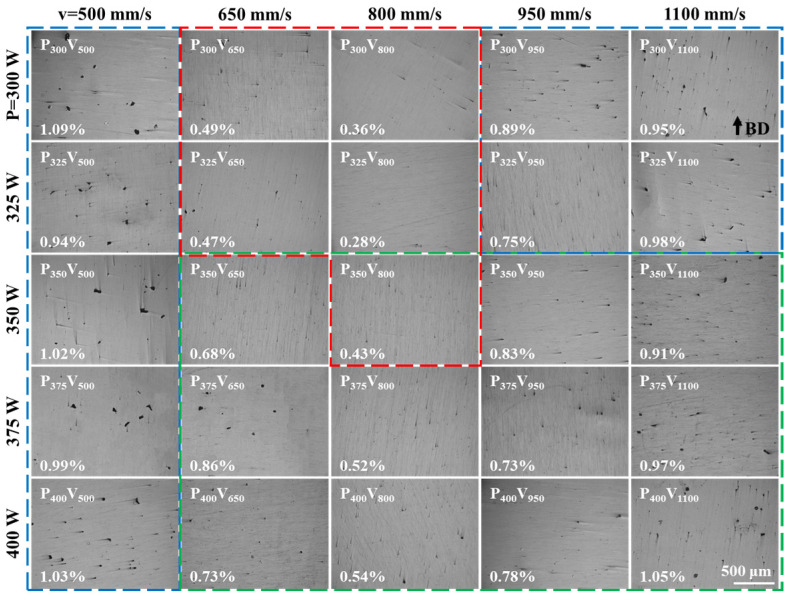
The metallographic microstructure of the XZ plane of the as-built samples (the lower-left corner shows the porosity, samples with a relative density higher than 99% are marked with a red box, as-built samples with irregular pores are marked with a blue box, and those with circular pores are marked with a green box).

Figure 3 shows micrographs of XZ planes (perpendicular to the direction of the substrate) of as-built Cu–Cr–Nb–Ti samples manufactured using different process parameters. The defects in as-built Cu–Cr–Nb–Ti samples are pores. No cracks are observed. The porosity of samples with a relative density higher than 99% (five samples in the red dotted box) is lower than 0.5% according to the statistics of the porosity of the as-built samples. At the same laser power, the porosity of the as-built samples manufactured at a scanning speed of 800 mm/s is the minimum. This result is consistent with the change in the relative densities of the as-built samples shown in Figure 2b. The Cu–Cr–Nb–Ti samples manufactured at low scanning speeds have mainly large irregular pores. The number and the size of pores in the samples prepared using the scanning speed of 800 mm/s are small. At high scanning speeds, the pore size and quantity increase again, consistent with the change in the relative density. The metallurgical defects in the as-built samples manufactured at a scanning speed of 500 mm/s are mainly large and irregular pores. As the scanning speed increases, the pore size decreases significantly. In the as-built samples manufactured at a scanning speed of 800 mm/s, no irregular pores are observed, and only a small number of round pores are observed. With a continuous increase in the scanning speed, the number of irregular pores in the samples manufactured using a low laser power increases, and the number of circular pores in the samples manufactured using a high laser power increases. When the scanning speed increases to 1100 mm/s, the number of irregular pores in the samples manufactured using laser powers of 300 W and 325 W increases, but the number of defects in the as-built samples is less than that in the as-built samples at the scanning speed of 500 mm/s. The defects in the as-built samples manufactured using laser powers of 350–400 W are still circular pores.

**Figure 4:**
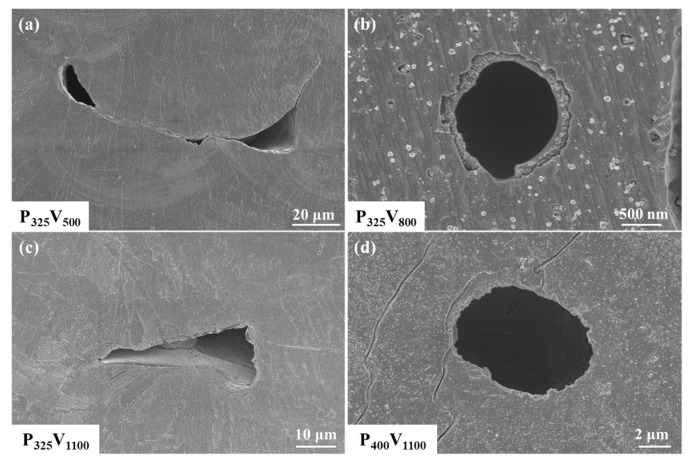
Defect morphology of Cu–Cr–Nb–Ti alloy prepared using different process parameters: (**a**) the irregular pore in sample P_325_V_500_, (**b**) the circular keyhole in sample P_325_V_800_, (**c**) the LOF pore in sample P_325_V_1100_, and (**d**) the keyhole in sample P_400_V_1100_.

The typical pore defects in the as-built samples in Figure 3 were observed by SEM, as shown in Figure 4. Figure 4a shows the irregular pores in sample P_325_V_500_. The pores are located at the overlapping position of the molten pool, with typical characteristics of lack-of-fusion (LOF) pores, but there is no unmelted powder inside the pores. Figure 4b shows the image of a pore in sample P_325_V_800_. The pore is nearly spherical, with typical keyhole characteristics and a diameter slightly larger than 1 μm. Figure 4c displays the image of an irregular pore in sample P_325_V_1100_. The irregular pore is located at the overlapping position of the molten pool, and there is unmelted powder in the pore, which also has the characteristics of a LOF pore. Figure 4d displays an enlarged image of the pore in sample P_400_V_1100_. The pore is oval and has typical keyhole characteristics. A comparison between Figure 4b,d shows that the average size of the keyhole increases with an increase in the laser power.

**Figure 5:**
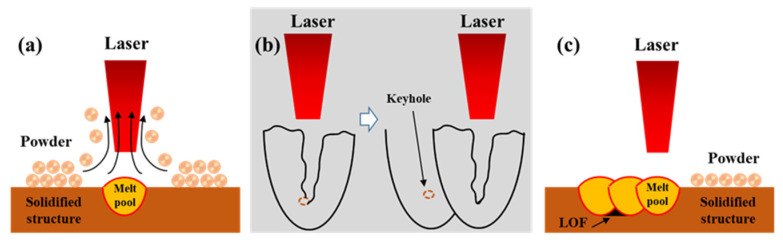
Schematic diagram of defect formation: (**a**) denudation, (**b**) keyhole, and (**c**) LOF pore.

The above results indicate that the formation of pores is closely related to SLM parameters, and the types and sizes of pores in as-built samples manufactured using different process parameters are quite different. The laser beam with a low scanning speed has a denudation effect on the powder under the Bernoulli effect [40] due to the presence of high-speed metal vapor flow above the molten pool. The powders around the molten pool are entrained and rolled out by the airflow, resulting in the loss of powder around the molten pool and insufficient alloy melt in the molten area, which creates irregular pores, as shown in Figure 5a. The denudation width decreases as the scanning speed increases [40]. Therefore, the amount of powder entrained and rolled out by the airflow around the molten pool decreases, and the powder loss in the molten zone decreases. This type of pore does not occur in samples with a high scanning speed. In addition, due to the high viscosity of the copper alloy, the Marangoni effect [41] is obvious during the process. A small amount of gas is drawn into the molten pool and forms circular keyholes, as shown in Figure 5b. As the scanning speed increases, using low laser power, the powders and solidified zone absorb less laser energy per unit of time, forming a narrow molten pool, and there are non-overlapping areas between adjacent molten pools, which form a small number of irregular LOF pores, and un-melted powder in the defects, as shown in Figure 5c. The samples prepared by a high scanning speed and high laser power have wider molten pools. The overlapping area of molten pools broadens, and no LOF pores are generated. Due to the Marangoni effect and alloy element evaporation, some gas is drawn into the molten pool, forming a circular keyhole.

### 3.2. Phase Constituent

**Figure 6:**
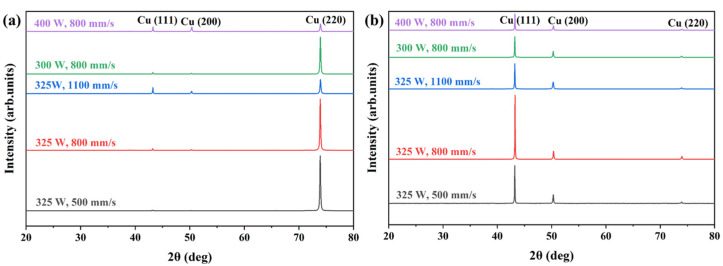
The XRD patterns of XY planes (**a**) and XZ planes (**b**) of Cu–Cr–Nb–Ti alloy manufactured using different process parameters.

Figure 6 shows the XRD patterns of XY and XZ planes of the Cu–Cr–Nb–Ti alloy samples prepared using different process parameters. Table 3 lists the results of the XRD analysis of Cu–Cr–Nb–Ti alloy. All samples only have diffraction peaks of FCC Cu (matrix phase), namely Cu (111), (200), and (220) peaks. Due to the low content of Cr, Nb, and Ti in the alloy, there are no diffraction peaks related to the second phase. The diffraction peaks of all as-built samples are shifted to the left, and the interplanar distances of all samples are greater than that of Cu, which indicates the solid solution of some Cr, Nb, and Ti atoms in the Cu matrix owing to the high cooling rate of SLM. The XRD patterns of XY planes (parallel to the direction of the substrate) of the samples prepared using different laser powers, and scanning speeds are displayed in Figure 6a. The peak strength of Cu (220) is the highest. The peak intensity of Cu (220) decreases as the scanning speed increases, while the peak intensities of Cu (111) and Cu (200) increase, indicating that increasing the scanning speed can weaken the degree of preferred orientation of grains in the samples prepared using the same laser power. When the scanning speed is 800 mm/s, the degree of grain-preferred orientation of the as-built sample manufactured using a laser power of 400 W is significantly weaker than that of the as-built sample manufactured using a laser power of 300 W. Figure 6b shows that the peak intensity of Cu (111) on the XZ plane is the highest and decreases as the scanning speed or the laser power increases, indicating that the degree of preferred orientation of grains in the samples decreases as the scanning speed or the laser power increases. However, the diffraction peak intensity of the XZ plane of the sample manufactured at a scanning speed of 800 mm/s is significantly higher than that of samples manufactured at other scanning speeds because the defects significantly reduce the diffraction peak intensity of the alloy. The sample manufactured using a scanning speed of 800 mm/s has the fewest defects and the highest relative density, so the diffraction intensity is the highest. The full width at half maxima (FWHM) of samples increases as the scanning speed or the laser power increases. This indicates that the grain size decreases as the scanning speed or the laser power increases.

### 3.3. Grain Morphology and Orientation

**Figure 7:**
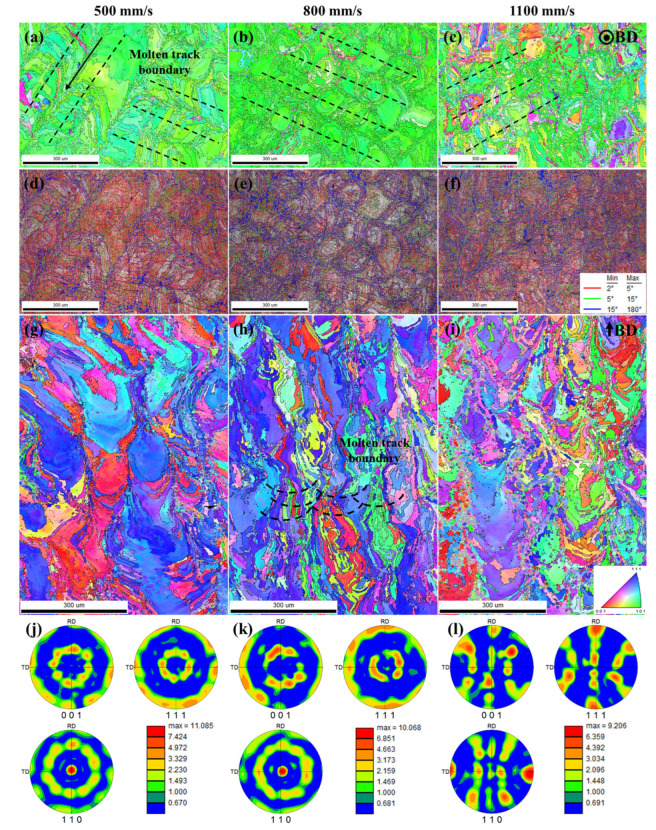
EBSD analysis results of Cu–Cr–Nb–Ti alloy samples manufactured by different scanning speeds (laser power of 325 W): (**a**–**c**) are IPFs of XY planes of samples manufactured by 500 mm/s (P_325_V_500_), 800 mm/s (P_325_V_800_), and 1100 mm/s (P_325_V_1100_) (the black dotted line is the boundary of the molten track, and the arrow is the laser moving direction); (**d**–**f**) are the IQ maps of XY planes of samples; (**g**–**i**) are IPFs of XZ planes of samples; (**j**–**l**) are the PFs of XY planes of samples.

To study the effect of scanning speed on the microstructure of Cu–Cr–Nb–Ti alloy, according to the analysis results in Figure 3 and Figure 6, samples manufactured using a laser power of 325 W at scanning speeds of 500, 800, and 1100 mm/s were selected for EBSD analysis, as shown in Figure 7. The inverse pole figures (IPFs) of XY planes of the samples show that XY planes of samples P_325_V_500_, P_325_V_800_, and P_325_V_1100_ have a bimodal grain size structure with alternating coarse grains (the size of grains greater than 10 μm) and fine grains. The coarse grains are shown as elongated and near-circular. As the scanning speed increases, the number of coarse grains decreases, and the number of fine grains increases. Figure 7d–f shows the image quality (IQ) maps of samples. There are obvious molten track boundaries in the samples, and the molten track width is about 100 μm. In the IPFs shown in Figure 7a–c, the molten track boundaries are marked with black dotted lines. The center of the molten track is fine-grained, both sides of the fine-grain area are coarse-grain areas, and the coarse grains grow along the direction of the laser beam movement. 

The IPFs of the XZ planes of the samples are displayed in Figure 7g–i. The microstructure of the XZ planes is composed of water-drop grains, long columnar grains, and equiaxed grains, and the number of equiaxed grains is small. With an increase in the scanning speed, the number of coarse grains decreases gradually but is still greater than that of equiaxed grains. In addition, the grains do not simply grow vertically along the build direction. The boundaries of the molten pools are represented by black dotted lines, as shown in Figure 7h. The grains are found to grow along the center of the molten pools. According to the grain morphology of the XZ plane, the fine grains on the XY plane of the alloy sample indicate the short axis of the long columnar grains, and the coarse grains on the XY plane indicate the cross-section of the water-drop grains or the long axis of the columnar grains.

Figure 7j–l shows the Pole Figures (PFs) of XY planes of the as-built samples. The grain orientation and texture strength of samples P_325_V_500_, P_325_V_800_, and P_325_V_1100_ were analyzed according to Figure 7a–c,g–l. As per Figure 7a,g,j, the XY plane grains of the sample manufactured using the scanning speed of 500 mm/s are mainly of a <101> texture, the maximum texture strength is 11.085, and the XZ plane exhibits mainly <001> and <111> textures. When the scanning speed increases to 800 mm/s, the <111> texture in the XY plane of the as-built sample increases, the maximum texture strength is 10.068, and the <101> texture in the XZ plane increases (see Figure 7b,h,k). The <001> texture and the <111> texture in the XY plane of the sample manufactured using a scanning speed of 1100 mm/s continue to increase, and the maximum texture strength is 9.206, while the <111> texture strength in the XZ plane decreases. In summary, with the increase in the scanning speed, the degree of grain-preferred orientation of as-built samples decreases, consistent with the analysis results of XRD.

**Figure 8:**
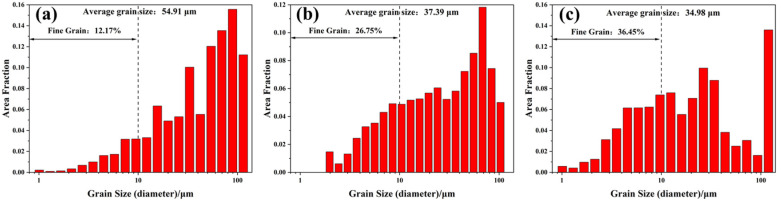
Statistical results of grain size on XY planes of Cu–Cr–Nb–Ti alloy samples manufactured by scanning speeds of (**a**) 500 mm/s (P_325_V_500_), (**b**) 800 mm/s (P_325_V_800_), and (**c**) 1100 mm/s (P_325_V_1100_) (laser power of 325 W).

Figure 8 shows the statistical analysis results of the grain size distribution on XY planes of samples P_325_V_500_, P_325_V_800_, and P_325_V_1100_. The average grain sizes of the samples are 54.91 μm, 37.39 μm, and 34.98 μm, respectively. The proportion of fine grains in samples is 12.17%, 26.75%, and 36.45%, respectively. This indicates that the average grain size of as-built Cu–Cr–Nb–Ti samples significantly reduce, and the proportion of fine grains significantly increases when the scanning speed increases.

**Figure 9:**
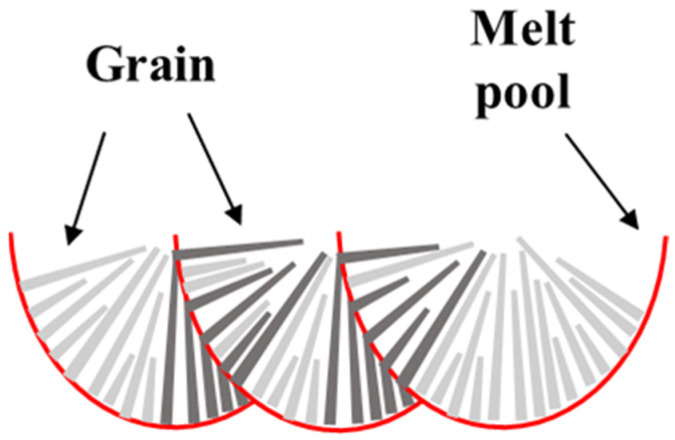
Schematic diagram of grain growth of the Cu–Cr–Nb–Ti alloy (dark gray grain represents the same grain).

In the SLM process, the temperature of the molten pool center is the highest, and the temperature of the molten pool edge is lower. Nucleation preferentially occurs at the edge of the molten pool and grows along the direction of the molten pool center. The center of the molten track on the XY plane comprises fine grains, and coarse grains are displayed on both sides of the fine-grain zone. The rotation angle of the adjacent as-built layer of SLM is 67°, and the overlapping of the adjacent molten pool leads to grain remelting, changing the direction of grain growth. The columnar grain grows along the thermal gradient direction (along the central area of the molten pool) rather than simply growing vertically along the build direction. Therefore, the grain morphology is more complex. Figure 9 shows the grain growth diagram. The grains grow along the thermal gradient direction. Hence, the lower the scanning speed, the longer the contact time between the powder and the laser beam per unit volume, the longer the heat-affected time on the samples being fabricated, the more obvious the preferred orientation of the grains, and the more prominent the <101> texture of the XY plane. As the scanning speed increases, the laser heat-affected time is shortened. This leads to increased undercooling, increased nucleation rate, and a reduced degree of preferred orientation of Cu–Cr–Nb–Ti alloy grains. Therefore, the <001> texture and the <111> texture in the XY plane increase, and the average grain size decreases.

**Figure 10:**
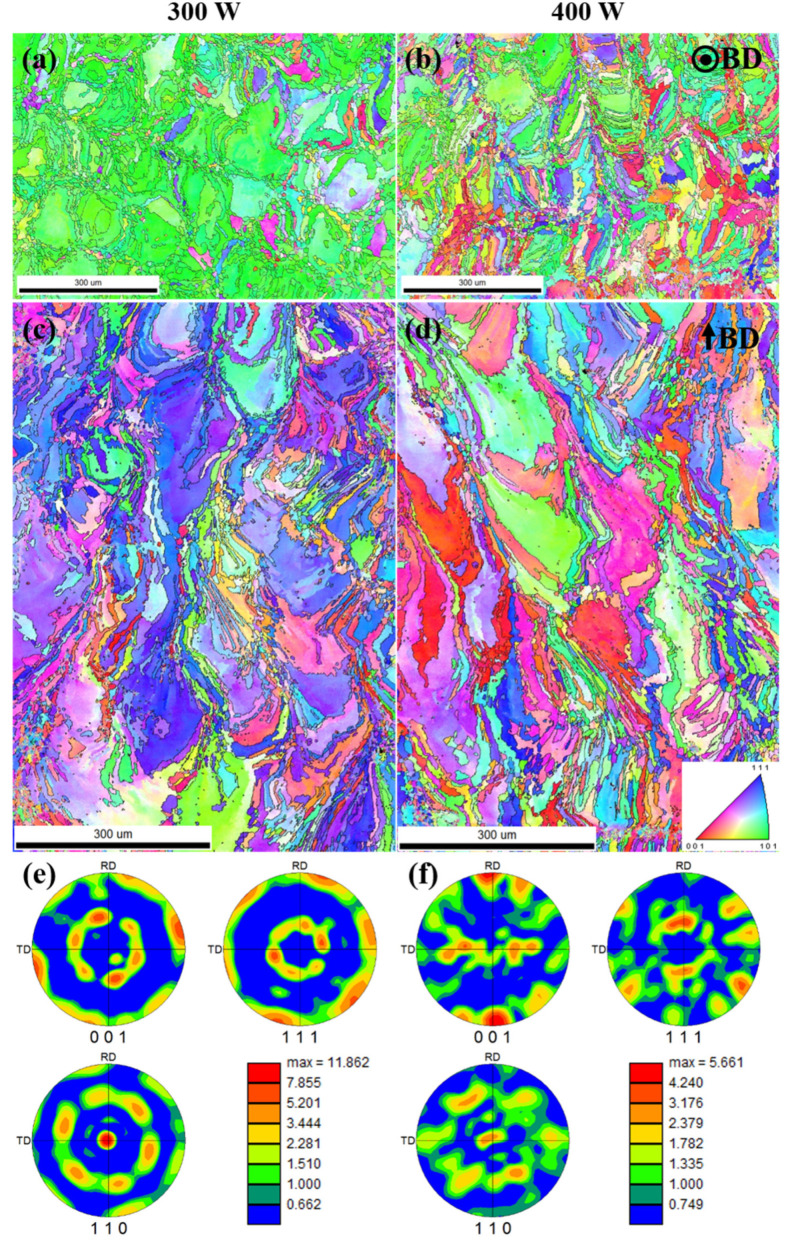
EBSD analysis results for Cu–Cr–Nb–Ti alloy samples manufactured using different laser powers (scanning speed of 800 mm/s): (**a**,**b**) are IPFs of XY planes of the samples manufactured using laser powers of 300 W (P_300_V_800_), and 400 W (P_400_V_800_), respectively; (**c**,**d**) are IPFs of XZ planes of samples; (**e**,**f**) are the PFs of XY planes of samples.

To analyze the effect of laser power on the grain morphology, grain orientation, and texture strength of the alloy, samples P_300_V_800_ and P_400_V_800_ manufactured at a scanning speed of 800 mm/s using laser powers of 300 W and 400 W, respectively, were selected for EBSD analysis, as shown in Figure 10. Figure 10a,b shows the IPFs of XY planes of the as-built samples. The maps show that XY planes of samples P_300_V_800_ and P_400_V_800_ have the same microstructure characteristics as those in Figure 7a–c; that is, they present an obvious bimodal grain size distribution with alternating coarse and fine grains. The grains are obviously refined as the laser power increases. The number of coarse grains on the XY plane of sample P_400_V_800_ is far less than that of sample P_300_V_800_. Specifically, the number of near-circular coarse grains significantly reduces. Figure 10c,d shows the IPFs of XZ planes of samples P_300_V_800_ and P_400_V_800_. The XZ plane is also composed of water-drop grains, long columnar grains, and equiaxed grains, and the grains grow along the center of the molten pool. Figure 10e,f shows the PFs of as-built samples. A comparison of Figure 10a,c,e with Figure 10b,d,f shows that the XY plane orientation of sample P_300_V_800_ exhibits mainly the <101> texture, with a texture strength of 11.862, and the <111> texture in the XZ plane is more prominent, while the XY plane of sample P_400_V_800_ has no obvious grain orientation, with a texture strength of 5.661, which indicates that the preferred orientation of the grains is more obvious in the sample manufactured using low laser power. Figure 10e,f shows that the XY plane of sample P_300_V_800_ exhibits the R-Goss {110}<110> texture [42], and the texture type of the XY plane of sample P_400_V_800_ has changed significantly, to the Goss {110}<100> texture.

**Figure 11:**
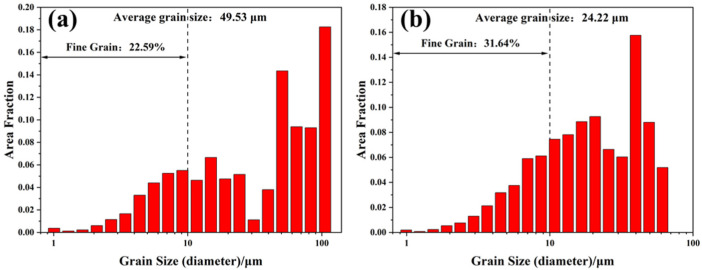
Statistical results of grain size on XY planes of Cu–Cr–Nb–Ti alloy samples manufactured using laser powers of (**a**) 300 W (P_300_V_800_) and (**b**) 400 W (P_400_V_800_) (scanning speed of 800 mm/s).

Figure 11 shows the grain size distribution of XY planes of samples P_300_V_800_ and P_400_V_800_. The average grain size of XY planes of samples P_300_V_800_ and P_400_V_800_ are 49.53 μm and 24.22 μm, respectively. The average grain size of sample P_400_V_800_ is 50% smaller than that of sample P_300_V_800_. The proportion of fine grains of samples P_300_V_800_ and P_400_V_800_ are 22.59% and 31.64%, respectively. This indicates that the average grain size of the samples decreases, and the proportion of fine grains increases with the increase in the laser power.

In the SLM process, with an increase in the laser power, the laser input energy increases, which will increase the depth and width of the molten pool, reduce the texture strength, and change the texture type. In addition, the nucleation rate of the melt increases owing to increased laser power and undercooling. Grains are obviously refined, and there is no obvious preferred orientation.

### 3.4. The Second Phase

**Figure 12:**
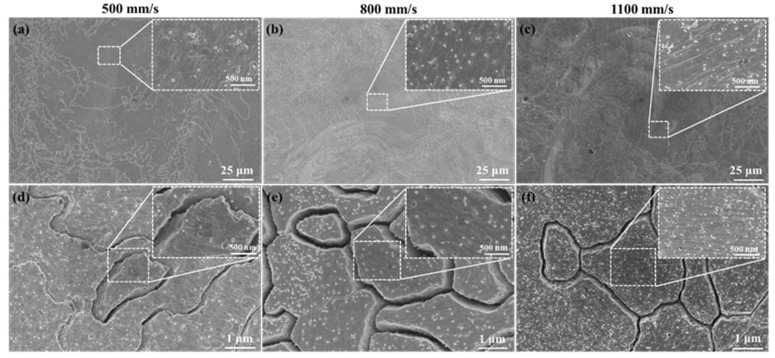
The second phases on the XY planes of Cu–Cr–Nb–Ti alloy samples manufactured at different scanning speeds using laser power of 325 W (the upper-right corner shows enlarged images): (**a**–**c**) the second phases in the coarse grains in the samples P_325_V_500_, P_325_V_800_, and P_325_V_1100_ manufactured by 500 mm/s, 800 mm/s, and 1100 mm/s, respectively; (**d**–**f**) are the second phases in the fine grains in samples.

**Figure 13:**
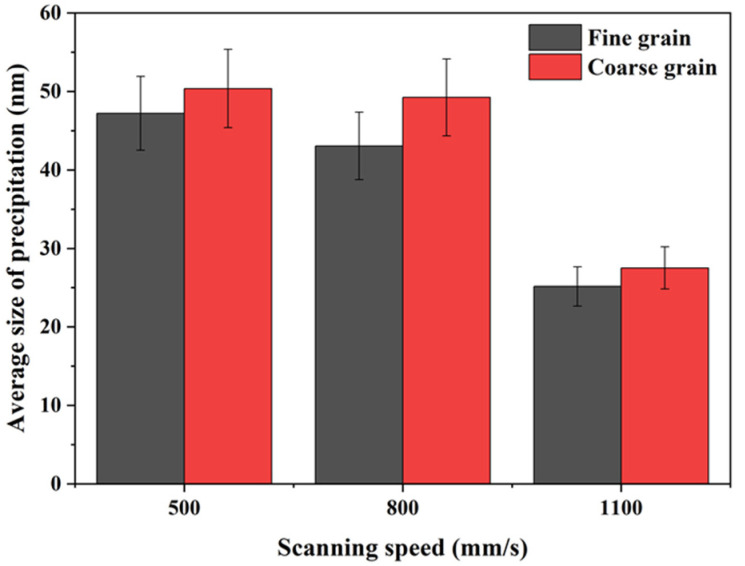
Statistical results of the size of the second phase in Cu–Cr–Nb–Ti alloy samples manufactured at different scanning speeds.

Figure 12 shows the SEM images of the second phase on XY planes of samples P_325_V_500_, P_325_V_800_, and P_325_V_1100_ manufactured using a laser power of 325 W at scanning speeds of 500, 800, and 1100 mm/s, respectively. Figure 13 shows the average size of the second phase. The second phase in the Cu–Cr–Nb–Ti alloy is fine and dispersed in the alloy grain. As the scanning speed increases, the number of second phases of as-built samples gradually increases, and the size decreases. The sizes of the second phase of samples manufactured using scanning speeds of 500 mm/s and 1100 mm/s are about 50 nm and 28 nm, respectively. In the same as-built sample, the sizes of the second phases in the fine grains are smaller than those of the second phases in the coarse grains. Due to the extremely high cooling rate of SLM, Cr, Nb, and Ti atoms dissolve in the copper matrix to form a supersaturated solid solution, and only a small amount of atoms form second phases. Therefore, the sizes of the second phase are significantly smaller than that of Cu–Cr–Nb alloys prepared using other processes, and there is no diffraction peak of the second phase in the XRD shown in Figure 6. With an increase in the scanning speed, the build time of the single layer of SLM samples is shortened, which increases the number of the second phase and decreases the size. The growth time of fine grains is shorter than that of coarse grains, and the second phase in fine grains has no time to grow, so the size of the second phase in fine grains is smaller than that in coarse grains.

**Figure 14:**
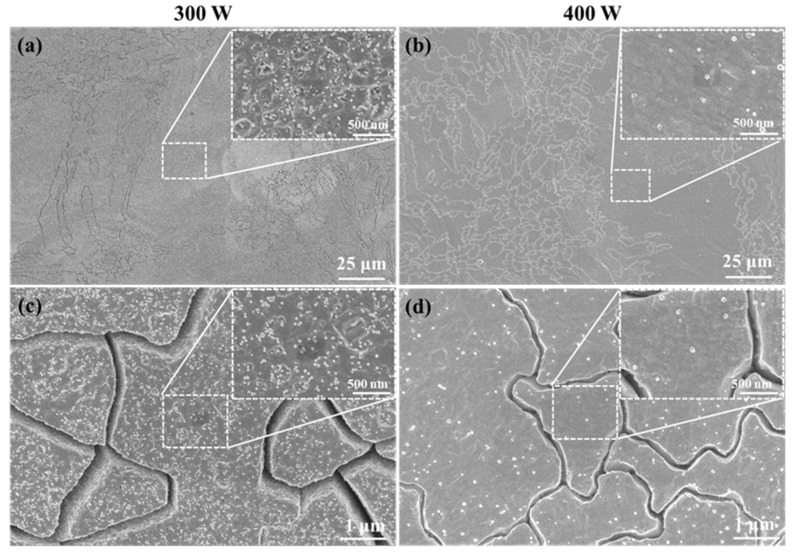
The second phase on the XY plane of Cu–Cr–Nb–Ti alloy samples manufactured using different laser powers at a scanning speed of 800 mm/s; the upper-right corner shows enlarged images: (**a**,**b**) are the second phases in the coarse grains in the samples P_300_V_800_ and P_400_V_800_ manufactured by 300 W and 400 W, respectively; (**c**,**d**) are the second phases in fine grains in samples.

Figure 14 shows the second phase morphology of XY planes of samples P_300_V_800_ and P_400_V_800_ manufactured at 800 mm/s using laser powers of 300 W and 400 W, respectively. Figure 14a,b shows the distribution of the second phases in coarse grains. Figure 14c,d shows the distribution of the second phases in fine grains. The Cu–Cr–Nb–Ti alloy samples manufactured using different laser powers have fine-sized second phases dispersed in the grain. As the laser power increases, the number of the second phase decreases significantly, and the size increases.

**Figure 15:**
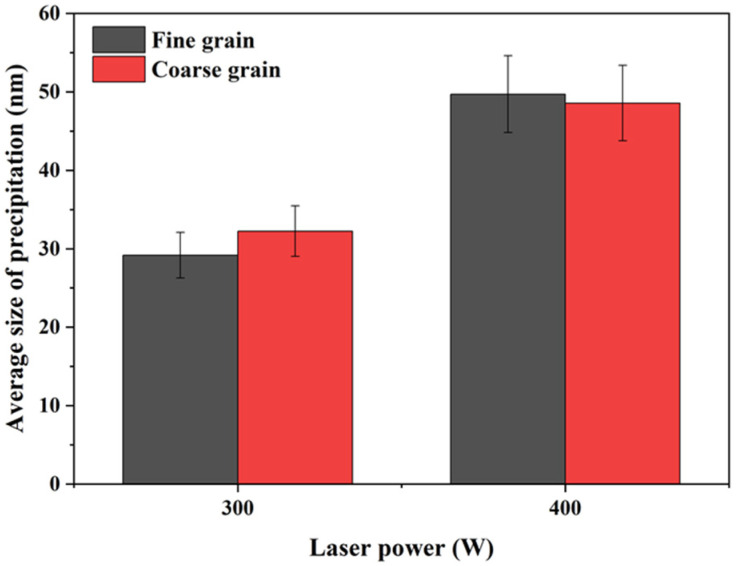
Statistical results of the size of the second phase in the Cu–Cr–Nb–Ti alloy samples manufactured using different laser powers.

Figure 15 shows the average size of the second phase in samples P_300_V_800_ and P_400_V_800_. The sizes of the second phases of samples manufactured using laser power of 300 and 400 W are about 30 nm and 50 nm, respectively. The sizes of the second phase in the fine grains are smaller than that in the coarse grains in the same as-built sample. The reason is that with an increase in the laser power, the laser input energy per unit volume increases, which increases the depth and width of the molten pools, increasing heat affected zone. This results in a longer growth time and a larger size of the second phase. In addition, due to the limited content of precipitated Cr and Nb elements, the number of the second phase is smaller when the laser power is higher.

### 3.5. Mechanical Properties and Electrical Conductivity

**Figure 16:**
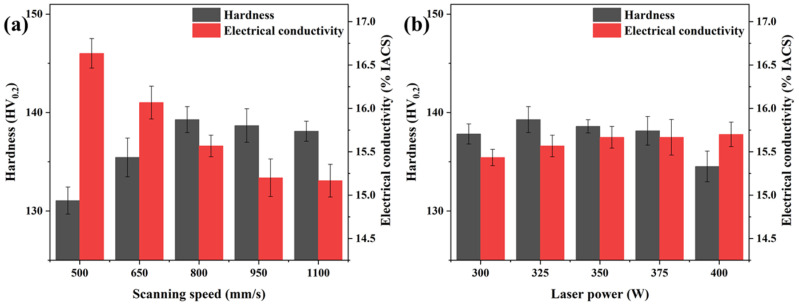
Effect of scanning speed (laser power of 325 W) (**a**) and laser power (scanning speed of 800 mm/s) on (**b**) the microhardness and electrical conductivity of as-built Cu–Cr–Nb–Ti alloy samples.

Figure 16 shows the effect of the process parameters on the microhardness and electrical conductivity of the Cu–Cr–Nb–Ti alloy. Figure 16a shows the microhardness and conductivity of the samples manufactured using different scanning speeds at the laser power of 325 W. With the increase in the scanning speed, the microhardness of the samples first increases and then decreases. The microhardness of the samples manufactured using scanning speeds of 800 mm/s and 500 mm/s are the highest (139 HV0.2) and the lowest (131 HV0.2), respectively. The electrical conductivity decreases with an increase in the scanning speed. The electrical conductivity of samples manufactured at scanning speeds of 500 mm/s, 800 mm/s, and 1100 mm/s are 16.6% IACS, 15.6% IACS, and 15.2% IACS, respectively. Figure 15b shows the microhardness and electrical conductivity of Cu–Cr–Nb–Ti alloy samples manufactured using different laser powers at the scanning speed of 800 mm/s. With an increase in the laser power, the microhardness of the samples first increases and then decreases, and the microhardness of the sample manufactured using a laser power of 325 W is the highest. With an increase in the laser power, the electrical conductivity of the samples increases slightly. The electrical conductivity of the samples manufactured using laser power of 300 W and 400 W are the lowest (15.4% IACS) and the highest (15.7% IACS), respectively.

**Figure 17:**
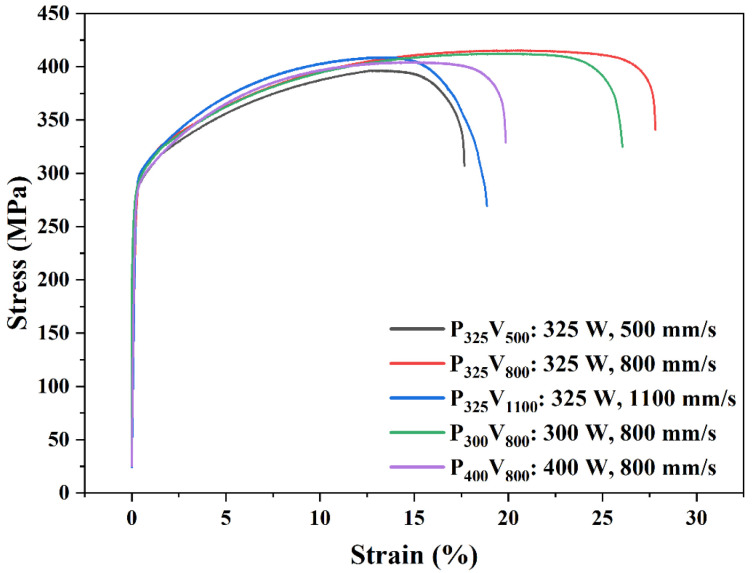
The stress-strain curve of Cu–Cr–Nb–Ti alloys manufactured using different process parameters.

Figure 17 shows the tensile stress-strain curve of as-built samples at room temperature. The corresponding tensile strengths and elongations are listed in Table 4. The mechanical properties of sample P_325_V_800_ are optimum. The tensile strength and elongation of P_325_V_800_ are 416 MPa and 27.8%. The tensile strength of the as-built Cu–Cr–Nb–Ti alloy is significantly higher than that of the as-built Cu–Cr–Nb alloy (382 MPa) [32]. Figure 16 and Figure 17 show the microhardness of as-built Cu–Cr–Nb–Ti alloy increases as the tensile strength increases.

The formula for calculating the strength of copper alloys *σ_b_* is [36]:

(2) $$\sigma_{b}=\sigma_{gs}+\sigma_{ss}+\sigma_{os}+\sigma_{dis}$$

where *σ_gs_* is strengthening by grain size reduction, *σ_ss_* is solid-solution strengthening, *σ_os_* is Orowan enhancement, and *σ_dis_* is dislocation strengthening. The SLM process has a high cooling rate, and as-built samples are supersaturated solid solutions, so the solid-solution strengthening and dislocation strengthening effects of samples manufactured using different process parameters are basically the same. Strengthening by grain size reduction is expressed using the Hall–Petch relation:

(3) $$\sigma_{gs}=\sigma_{0}+kd^{-\frac{1}{2}}$$

where *σ*_0_ is the friction stress of the copper alloy, *k* is the Hall–Petch constant (0.15), and *d* is the grain size. As the scanning speed increases, the grain size of the alloy decreases, resulting in strengthening by grain size reduction. In addition, there is a fine dispersed second phase in as-built samples, which can hinder the movement of dislocation, increase *σ_os_*, and increase the strength of the alloy. However, due to many defects in samples prepared using high scanning speed, the properties of the alloy reduce, so the microhardness and tensile strength of the alloy prepared using a scanning speed of 800 mm/s are the highest. The higher the laser power, the finer the grain of the as-built sample, and the smaller the number of the second phase. The strengthening by grain size reduction increases while the Orowan-strengthening decreases. Therefore, there is no obvious change in the microhardness and tensile strength of the alloy. Among as-built samples prepared using different laser powers, the samples prepared using laser power of 325 W have the fewest defects, so the microhardness and tensile strength of samples are the highest.

The formula for calculating the resistivity *ρ* of copper alloys is:

(4) $$\rho =\rho_{pho}+\rho_{def}+\rho_{int}+\rho_{imp}$$

where *ρ_pho_* is the phonon scattering resistance, *ρ_def_* is the scattering resistance caused by the defect, *ρ_int_* is the scattering resistance caused by the interface, and *ρ_imp_* is the impurity scattering resistance. At room temperature, the effect of *ρ_pho_* is negligible. As the scanning speed increases, the relative density of the alloy first increases and then decreases, and the proportion of defects first decreases and then increases. Therefore, *ρ_def_* first increases and then decreases. The average grain size in the alloy decreases, the number of fine grains increases, and the scattering resistance *ρ_int_* caused by the interface increases continuously. The number and size of the second phase increase, and the scattering ability of electrons increases so *ρ_imp_* increases. Therefore, the electrical conductivity of the Cu–Cr–Nb–Ti alloy decreases as the scanning speed increases. The laser power increases, and the grain size of the alloy reduces, increasing *ρ_int_*. In addition, the number of the second phase decreases, the second phase size increases, and *ρ_imp_* keeps decreasing. The two scattering resistance cancel each other out, so the electrical conductivity hardly changes.

## 4. Conclusions

In this article, the effects of SLM process parameters on the relative density, microstructures (such as defects, grain morphology, and orientation, as well as the size and distribution of the second phase), mechanical properties, and electrical conductivity of the Cu–Cr–Nb–Ti alloy were studied. The following conclusions can be drawn:

(1) As the laser power or the scanning speed increases, the relative density of as-built Cu–Cr–Nb–Ti alloy first increases and then decreases. The as-built sample prepared using a laser power of 325 W and a scanning speed of 800 mm/s has the least defects and the highest relative density. The defects in the samples with scanning speeds lower than 800 mm/s are mainly LOF pores caused by denudation. When the speed is higher than 800 mm/s, the alloy samples manufactured using a low laser power have LOF pores due to low input energy, and the samples manufactured using a high laser power have keyholes. The VED can only be used as a reference parameter for manufacturing a SLMed Cu–Cr–Nb–Ti alloy.
(2) The SLMed Cu–Cr–Nb–Ti alloy only has diffraction peaks of FCC Cu (matrix phase). The diffraction peaks shift to small angles, and the interplanar distances are greater than that of Cu. The diffraction peaks related to the second phase are not observed. The degree of grain-preferred orientation of as-built samples decreases as scanning speed or laser power increases. FWHM of samples increases as the scanning speed or the laser power increases. The intensity of Cu peaks of as-built alloy manufactured using 325 W, and 800 mm/s is the highest.
(3) The XY plane of the Cu–Cr–Nb–Ti alloy is composed of fine grains in the center of the molten track and coarse grains on both sides. The microstructure of the XZ plane is composed of water-drop grains, long columnar grains, and equiaxed grains. The average grain size of XY planes of all samples is in the 24–55 μm range. With an increase in the scanning speed or the laser power, the proportion of fine grains in the XY plane increases, the average grain size decreases, and the degree of preferred orientation of grains decreases. The texture type of the XY plane of the SLMed alloy changes from R-Goss texture to Goss texture as laser power increases.
(4) Cu–Cr–Nb–Ti alloy has fine and dispersed second phases with a size of 28–50 nm. As the scanning speed increases, the size of the second phase decreases, but the number increases. When the laser power increases, the size of the second phase increases, but the number decreases. The size of the second phase is smaller in fine grains than in coarse grains.
(5) The microhardness, tensile strength, and elongation of a Cu–Cr–Nb–Ti alloy first increase and then decrease as scanning speed or laser power increases. The electrical conductivity decreases with increasing scanning speed and increases with increasing laser power. The Cu–Cr–Nb–Ti alloy manufactured using the optimum process parameters of 325 W, and 800 mm/s has the highest microhardness, tensile strength, and elongation, namely 139 HV_0.2_, 416 MPa, and 27.8%, respectively, and the electrical conductivity is 15.6% IACS. The mechanical properties of the SLMed Cu–Cr–Nb–Ti alloy are significantly higher than those of the SLMed Cu–Cr–Nb alloy.

## Figures and Tables

**Table 1:** The chemical composition of the Cu–Cr–Nb–Ti alloy powders.

Element	Cr	Nb	Ti	Cu
Measured Composition (wt. %/at. %)	1.65/2	1.35/0.92	0.12/0.2	Balance

**Table 2:** Sample number and corresponding laser power and scanning speed.

	500 mm/s	650 mm/s	800 mm/s	950 mm/s	1100 mm/s
300 W	P_300_V_500_	P_300_V_650_	P_300_V_800_	P_300_V_950_	P_300_V_1100_
325 W	P_325_V_500_	P_325_V_650_	P_325_V_800_	P_325_V_950_	P_325_V_1100_
350 W	P_350_V_500_	P_350_V_650_	P_350_V_800_	P_350_V_950_	P_350_V_1100_
375 W	P_375_V_500_	P_375_V_650_	P_375_V_800_	P_375_V_950_	P_375_V_1100_
400 W	P_400_V_500_	P_400_V_650_	P_400_V_800_	P_400_V_950_	P_400_V_1100_

**Table 3:** The XRD analysis results of Cu–Cr–Nb–Ti alloy manufactured using different process parameters.

	P_325_V_500_ XY	P_325_V_800_ XY	P_325_V_1100_ XY	P_300_V_800_ XY	P_400_V_800_ XY	P_325_V_500_ XZ	P_325_V_800_ XZ	P_325_V_1100_ XZ	P_300_V_800_ XZ	P_325_V_500_ XY
2θ	73.901	73.902	73.942	73.923	73.942	43.199	43.244	43.202	43.201	43.222
d	1.2814	1.2814	1.2808	1.2811	1.2808	2.0925	2.0904	2.0924	2.0924	2.0914
Height	88,994	166,388	22,744	60,152	12,068	176,175	598,444	117,784	96,658	75,968
Area	983,543	1,809,151	272,793	681,089	149,904	1,027,731	3,298,966	763,149	651,673	449,052
FWHM	0.188	0.185	0.204	0.192	0.211	0.094	0.099	0.110	0.100	0.115
Degree of crystallinity	95.06%	96.83%	96.26%	96.24%	96.28%	96.56%	97.54%	96.56%	96.54%	96.38%

**Table 4:** Mechanical properties of as-built Cu–Cr–Nb–Ti alloys manufactured using different process parameters.

Samples	Laser Power (W)	Scanning Speed (mm/s)	Microhardness (HV0.2)	Tensile Strength (MPa)	Elongation (%)
P_325_V_500_	325	500	131 ± 1	396 ± 6	17.7 ± 2.2
P_325_V_800_	325	800	139 ± 1	416 ± 5	27.8 ± 2.8
P_325_V_1100_	325	1100	138 ± 1	404 ± 3	19.9 ± 3.6
P_300_V_800_	300	800	138 ± 1	412 ± 3	26.1 ± 3.2
P_400_V_800_	400	800	135 ± 2	403 ± 4	20.8 ± 2.2

## Data Availability

The data presented in this study are available on request from the corresponding author.
